# The jaws in the jaws: Morphofunctional analysis of the pharyngeal plates of *Labrus viridis* Linnaeus, 1758 (Teleostei, Labridae)

**DOI:** 10.1111/jfb.70536

**Published:** 2026-06-14

**Authors:** Cristina Gioia Di Camillo, Gaspare Zichittella, Francesco Tiralongo

**Affiliations:** ^1^ Department of Life and Environmental Sciences Polytechnic University of Marche Ancona Italy; ^2^ National Biodiversity Future Center (NBFC) Palermo Italy; ^3^ Consorzio Nazionale Interuniversitario per le Scienze del Mare (CoNISMa) Rome Italy; ^4^ Scientific Illustrator, San Giovanni Teatino Chieti Italy; ^5^ Department of Biological, Geological, and Environmental Sciences University of Catania Catania Italy; ^6^ Ente Fauna Marina Mediterranea Avola Italy; ^7^ National Research Council, Institute of Marine Biological Resources and Biotechnologies Messina Italy

**Keywords:** durophagy, gut contents, jaw apparatus, pharyngognathous fishes, plicidentine, polyphyodonty

## Abstract

Functional morphology highlights the adaptive flexibility of fish feeding strategies across environmental gradients and seasonal variations, providing key insights for the management and conservation of ichthyofaunal communities and their habitats. Labridae fishes possess a specialized pharyngeal jaw apparatus (PJA) that enables them to crush and consume prey with heavily calcified or chitinized shells. This functional adaptation has expanded their dietary flexibility, promoted trophic specialization and enhanced the ecological diversity of wrasses. Here, we present the first comprehensive morphological analysis of the pharyngeal jaws of *Labrus viridis* Linnaeus, 1758, a labrid fish widely distributed in shallow coastal habitats of the Mediterranean Sea. The PJA was examined using micro‐computed tomography (micro‐CT), scanning electron microscopy and energy‐dispersive X‐ray spectroscopy. Stomach contents from eight specimens were also analysed to infer prey composition and feeding strategy. The pharyngeal jaws of *L. viridis* are characterized by unicuspid molariform and caniniform teeth, showing intraosseous tooth development, weakly developed plicidentine and a one‐to‐one tooth replacement. This heterodont dentition, together with the predominance of caniniform over molariform teeth and the diverse prey items identified in gut contents, indicates an opportunistic feeding strategy and a diet distinct from that of the congeneric *Labrus bergylta*. This work suggests the existence of ecological divergence among congeneric species. Moreover, our results provide a reference point for linking the morphofunctional traits to the dietary role of *L. viridis*, encouraging new investigations into the morphology of the PJA and trophic dynamics of other Mediterranean wrasses.

## 1 INTRODUCTION

Labridae family constitutes one of the most species‐rich families of marine fishes (more than 550 species), displaying a remarkable diversity in coloration, body shape, cranial structure and jaw morphology (Parenti & Randall, 2018; Westneat et al., 2005). This morphological diversity reflects their wide range of feeding behaviour and dietary specializations (Clifton & Motta, 1998) and highlights how innovative functional traits spurred a widespread adaptive radiation (Burress et al., 2019).

Beyond the oral jaws, Labridae exhibit a specialized set of throat‐based jaws called the pharyngeal jaw apparatus (PJA), a trait known as pharyngognathy (Evans et al., 2019; Liem, 1973; Wainwright et al., 2012). The pharyngeal jaws derive from modifications of pharyngeal arches and consist of a couple of distinct upper plates (left and right) and two fused lower bones forming a unique plate connected to the neurocranium by a muscular sling (Mabuchi et al., 2007). More specifically, the upper and the lower pharyngeal plates correspond to the third pharyngobranchials (PB3) and the fused fifth ceratobrachials (CB5), both equipped with stout conical teeth (Liem & Sanderson, 1986). The masticatory orbits described by the lower and upper jaws apparatus during feeding together with the force generated by the pharyngeal muscles facilitate both shearing and crushing of prey (Liem & Sanderson, 1986). Thanks to the PJA and the strong bite forces exerted by the branchial musculature, Labridae fish can crush and feed on heavily calcified or chitinized shells, a feeding behaviour known as durophagy (Fruciano et al., 2011; Grubich, 2003). Indeed, the diet of Labridae includes algae and seagrasses, foraminifers, mussels, gastropods, chitons, crustaceans including barnacles, echinoids, tunicates, polychaetes, amphipods and other fish (Beltrano et al., 2006; Ben Slama et al., 2007; Dipper et al., 1977; Dulčić, 1999; Kabasakal, 2001; Liem & Sanderson, 1986).

Pharyngeal jaws function as secondary jaws, anatomically and functionally distinct from the oral ones. The anatomical separation between oral and pharyngeal jaws enabled labrid fish to differentiate the functions of prey capture and food transport to the oesophagus (Liem, 1973; Mabuchi et al., 2007). The lips of Labridae are used to draw in prey, which is then ground down by the PJA (Dipper et al., 1977). These traits expanded dietary flexibility, promoting trophic specialization and enhancing ecological diversity of Labridae (Wainwright, 2005). Pharyngognathy was pivotal in the diversification of several fish families, among which Cichlidae, Labridae and Pomacentridae, even though we are still far from fully understanding the complex evolutionary dynamics of this trait (Borstein et al., 2024). Considering the cranial architecture, the labrid cranium is a highly complex, multi‐element structure, exhibiting clear evolutionary modularity, allowing cranial modules to follow quasi‐independent evolutionary trajectories (Larouche et al., 2023). Within this framework, the module represented by the oral and the pharyngeal jaws, despite distinct developmental origins, shows strong evolutionary integration and have coevolved more tightly with each other than with other cranial elements, likely reflecting their sequential roles in feeding, with the oral jaws capturing prey and the pharyngeal jaws subsequently manipulating food (Evans et al., 2023; Larouche et al., 2023).

Besides providing insights on fish trophic ecology (Kabasakal, 2001), the size and shape of the pharyngeal jaws, along with the number and arrangement of their teeth, are key diagnostic features (Tigano et al., 1999).

Twenty‐two species of Labridae and have been identified in the Mediterranean Sea (Kovačić et al., 2021), four of which belonging to the genus *Labrus* Linnaeus, 1758 (*L. viridis* Linnaeus, 1758, *L. bergylta* Ascanius, 1767, *L. merula* Linnaeus, 1758 and *L. mixtus* Linnaeus, 1758). To date, descriptions of pharyngeal jaws in Mediterranean *Labrus* spp. exist only for *L. bergylta* Ascanius, 1767, based on fish collected outside the basin (Collins & Underwood, 2021; Dipper et al., 1977; Fries et al., 1892). A combination of traditional and geometric morphometrics was used to detect variability in the lower jaw of *Coris julis* (Linnaeus, 1758) from Eastern Sicily (Fruciano et al., 2011), while 3D geometric morphometric has been used to study shape variations in the cranial bones across Labridae (Evans et al., 2023; Larouche et al., 2023).

In any case, this suggests that the morphological traits of the pharyngeal jaws of most Mediterranean wrasses, and consequently their functional diversity (Westneat et al., 2005), are still far from being fully understood. A detailed analysis and description of the pharyngeal jaws, combined with data on diet and body size, would be essential for accurately determining prey variety and size and for defining the ecological role played by Labridae (Cardozo‐Ferreira et al., 2018).

In addition, examining functional morphology sheds light on the adaptive flexibility of fish feeding strategies in response to environmental gradients and seasonal variations, providing key information for the management of ichthyofaunal communities and their habitats (Schoenfuss & Blob, 2007). Understanding fish functional morphology is increasingly recognized as essential for advancing conservation efforts (Sun et al., 2025).

This research is particularly urgent, given that thermal stressors, invasive species, habitat degradation and overfishing could alter prey composition and availability and potentially lead to shifts in distribution and abundance in Labridae populations (Matić‐Skoko et al., 2013; Parker et al., 2019; Torreblanca & Báez, 2025).

*L. viridis*, also known as green wrasse, is a labrid fish (subfamily Labrinae) typically associated with rocky habitats (Kožul et al., 2011) and seagrass meadows (Lattanzi et al., 2024; Tiralongo et al., 2021) and distributed across the Mediterranean and Black Seas, as well as along the Atlantic coasts of Portugal and Morocco (FishBase). It has been classified as Vulnerable in the IUCN Red List of Threatened Species and its population trend is ‘Decreasing’; it is a species with potential for rearing, both for commercial and restocking purposes (Kožul et al., 2011).

The main objectives of this work are to (1) provide a detailed morphological description of the pharyngeal jaws of *L. viridis*; (2) offer new insights into the ultrastructure and polyphyodonty of labrids' PJA using scanning electron microscopy, energy‐dispersive X‐ray spectroscopy (EDS) and micro‐computed tomography (micro‐CT) techniques; and (3) enhance our understanding of the relationship between the jaw apparatus, diet and the trophic role of this species.

This study will establish a reference point for linking the morphofunctional traits to the dietary role of this species, encouraging new investigations into the morphology of the PJA and trophic dynamics of other Mediterranean wrasses. These data would inform management strategies and conservation aquaculture practices (Froehlich et al., 2017).

## 2 MATERIALS AND METHODS

### 2.1 Fish collection and dissection

First record of pharyngeal teeth of *L. viridis* was made by G. Zichittella while cleaning a specimen caught during a spearfishing trip. To examine and describe the pharyngeal plates, another specimen of *L. viridis* was caught by G. Zichittella on 05‐04‐2025 by spearfishing near Marsala (Sicily, Italy) at a depth of 2.7 m on *Posidonia oceanica* meadows; details of this last record were outlined in a standardized spreadsheet (Table S1) following Di Camillo et al. (2018). The fish was promptly placed on ice and subsequently frozen at −20°C to preserve its morphological features and gut contents. Before dissecting the fish and collecting the pharyngeal jaws, the fish was photographed at different angles and magnification using a reference scale to obtain morphometric data. Then, the operculum bone and the first four gill filaments were removed using a scalpel and forceps to take pictures of the last branchial arches bearing the plates. After collecting the pharyngeal plates, dissected parts were boiled for 2 min to remove fish tissue; then, the plates were gently separated from flesh and cleaned with a natural bath sponge piece under a stereomicroscope to remove small flesh remnants.

Additionally, seven specimens of *L. viridis* with TL (total length) range of 19–32 cm collected in the Gulf of Catania (Sicily, Ionian Sea) by fishermen between August and September 2025 at depth 2–15 m were studied for stomach content composition.

To examine fish gut contents, a ventral incision was made from the anus to the gills to expose and extract the stomach.

### 2.2 Scientific drawings

Scientific artwork depicting the fish and its oral and pharyngeal jaws was created through a combination of pencil, ink pen and acrylic painting techniques. The drawings include full‐body views of the fish and transparent renderings of the head, highlighting the jaws in lateral and frontal views.

### 2.3 Electron microscopy, EDS and microtomography

To study the ultrastructure and to acquire micrographs of the pharyngeal plates, the samples were washed with distilled water and dehydrated in a graded ethanol series. Successively, the jaws were coated with gold–palladium in a Balzer Union evaporator and examined with a TESCAN VEGA‐III scanning electron microscope (TESCAN, a.s., Brno, Czech Republic) operated with a beam of 20 kV in a high vacuum (HV) mode. The morphological characterization was combined with a chemical surface analysis conducted through EDS (OXFORD INCAx‐act) to identify the elements in the plates.

To investigate the micro‐anatomy of the pharyngeal jaws and teeth, 3D reconstructions of upper and lower plates were obtained through X‐ray micro‐CT, using a desktop device Bruker SkyScan 1174 μ‐CT system (SkyScan‐Bruker, Antwerp, Belgium) set with an acceleration voltage of 50 kV, beam current of 800 μA; aluminium filter (thickness of 1 mm); rotation of 180° in steps of 0.2°; time per projection equal to 8 s. For the upper plate the pixel size was set to 7.6 μm, for the lower to 22.2 μm. NRecon software (version 1.6.10.2, Bruker, Billerica, MA, USA) was used to convert the projections into slices, setting the following parameters: smoothing: 5%; ring artefact removing: 1; beam hardening correction: 40%.

Measurements of the plates (maximal length, height and width of the dentigerous bone; area of the toothed side) and teeth (height and maximal diameter) were determined from the collected micrographs using the free software ImageJ (Schneider et al., 2012).

Artificial Intelligence was used exclusively to check the accuracy of some English sentences.

## 3 RESULTS

### 3.1 The collected specimens of *L. viridis*

**FIGURE 1:**
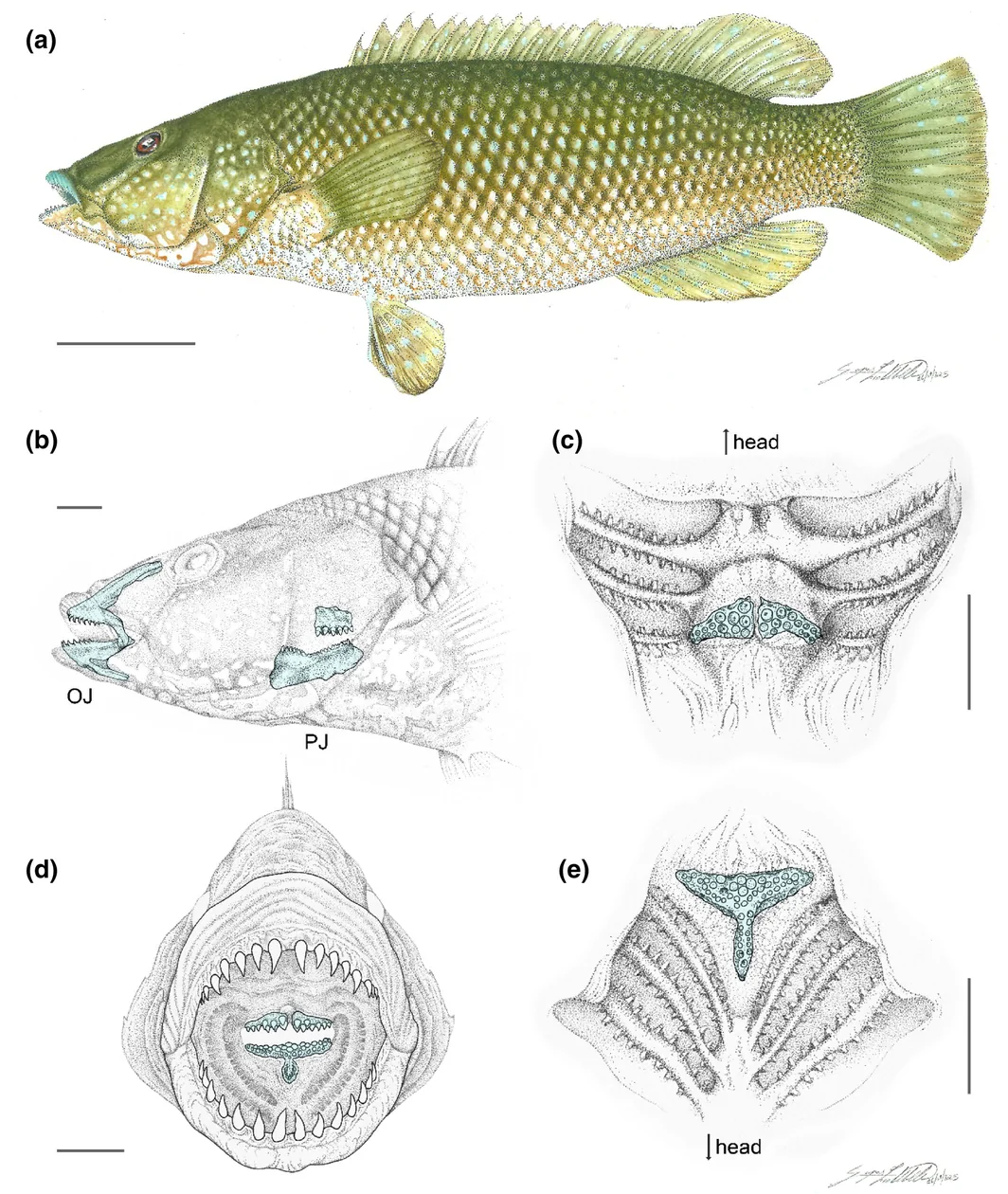
(a) Illustration of the collected specimens of *Labrus viridis* from Marsala. Drawing of Gaspare Zichittella, 16‐09‐2025. Scale bar 5 cm. (b–e). Diagrams showing the position of the pharyngeal jaw apparatus in the head of *L. viridis* viewed from different perspectives. Pharyngeal plates are highlighted in cyan. Lateral (b) and frontal (d) view of the fish head showing oral (OJ) and pharyngeal jaws (PJ). (c) Upper right (R) and left (L) plates. (e) Lower plate. Drawings of Gaspare Zichittella, 16‐09‐2025. Scale bar 5 cm.

The specimen captured in Marsala has preserved its morphological characteristics and typical coloration intact (Figure 1a). The fish measured 35.5 cm (total length), 31 cm (standard length); head length (from mouth to operculum edge) 9.1 cm; body depth: 7.9 cm; snout length: 3.3 cm.

Gut contents consisted of a Syngnathidae of about 19 cm and a fragment of seagrasses, likely belonging to Cymodoceaceae or to Zosteraceae.

Among the seven specimens collected in the Gulf of Catania, two had empty stomachs and one contained highly digested, unidentifiable material. The remaining four individuals each presented distinct prey items: one contained remains of the crab *Eriphia verrucosa* (Forskål, 1775); another showed damaged, unidentifiable shrimp remains; a third had three amphipods along with fragments of bivalve shells; and the last specimen contained chiton remains.

### 3.2 The pharyngeal jaws

The PJA is composed of two truncated pyramidal upper plates and one trilobate lower plate (LTP) exhibiting heterodont dentition with unicuspid teeth. The PJA is positioned inside the fish head as presented in Figure 1b–e. All the plates were fully embedded in living tissue, with only the teeth protruding (Figure S1).

**FIGURE 2:**
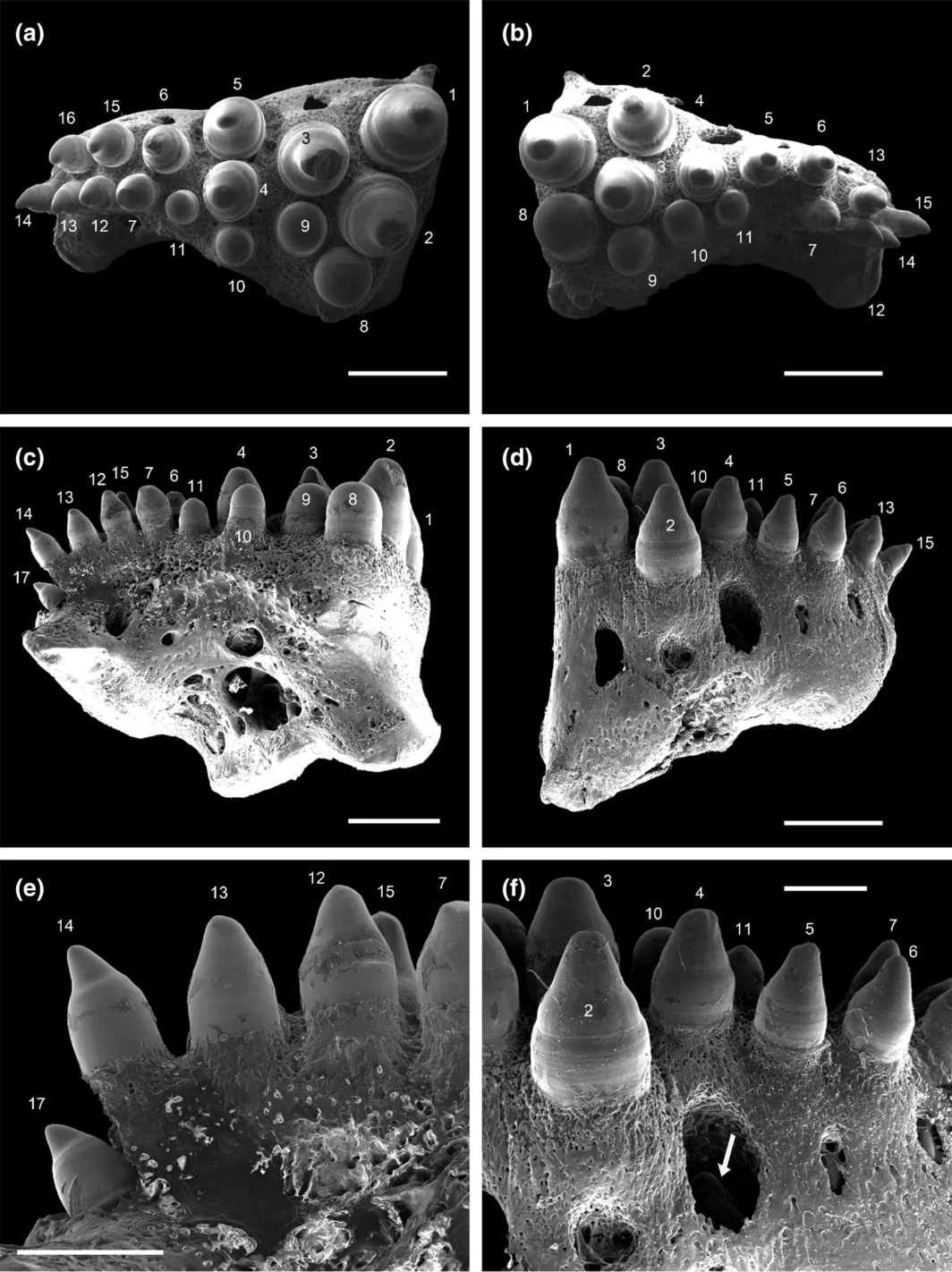
Scanning electron microscope photographs of upper right (a, c, e) and left (b, d, f) plates composing the pharyngeal jaws apparatus of *Labrus viridis*. Upper (a, b) and lateral (c, d) view of the right (a, c) and the left (b, d) plates. Note the central largest caniniform teeth lying in a depression of the upper right plate (a). (e, f) Particular of the teeth of the right (e) and left (f) plates with a germ tooth visible from one of the holes (arrow). Scale bars: a–d: 2 mm; e–f: 1 mm.

The upper right plate (URP) measures 7.4 mm (length) × 5.4 mm (width) × 1.7 mm (height) and shows a toothed side area of 28.3 mm^2^ with 17 teeth (Figure 2a,c,e, Table 1).

**TABLE 1:** Types and size of teeth.

Plate	Tooth type	n.	Size (height × diameter)	
Upper right (toothed side area of 28.3 mm^2^ with 17 teeth)	Caniniform (1–7)	7	1.0–1.9 mm × 0.6–1.3 mm	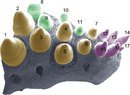
	Molariform (8–11)	4	0.6–1.5 mm × 0.5–1.1 mm	
	Sharply pointed caniniform (12–17)	6	0.7–1.8 mm × 0.5–0.7 mm	
Upper left (toothed side area of 24.4 mm^2^ with 15 teeth)	Caniniform (1–7)	7	1.3–2.3 mm × 0.9–1.6 mm	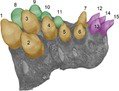
	Molariform (8–11)	4	0.9–1.5 mm × 0.5–1.3 mm	
	Sharply pointed caniniform (12–15)	4	0.7–0.9 mm × 0.5–0.6 mm	
Trilobate lower (toothed side area of 40.3 mm^2^ with 48 teeth + 3 likely worn‐down)	Caniniform (1–24)	23	0.8–1.7 mm × 0.5–0.9 mm	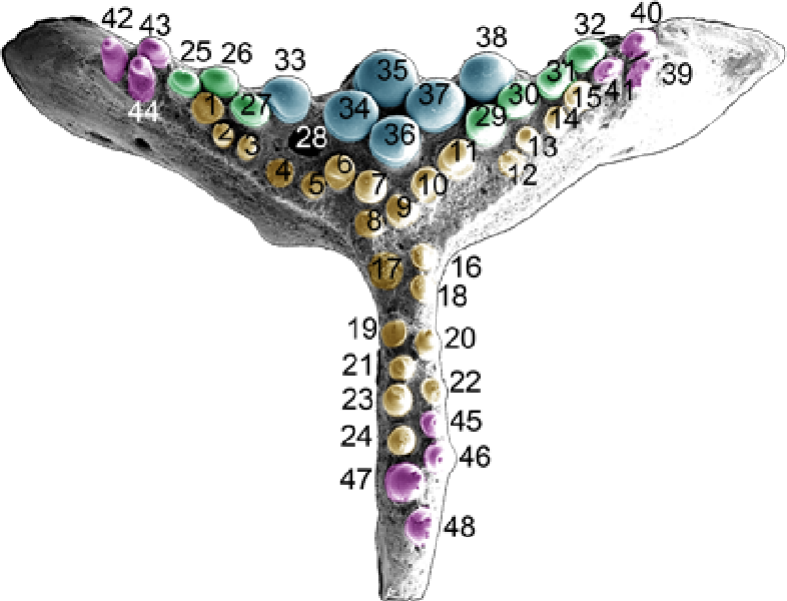
	Small molariform (25–32)	8	1.2 mm × 0.8–1.1 mm	
	Large molariform (33–38)	6	1.8–2.1 mm × 1.4–1.7 mm	
	Sharply pointed caniniform (39–48)	10	1.1–1.8 mm × 0.6–0.7 mm	

*Note*: The digitally recoloured photographs on the right show the different tooth types (green: molariform, cyan: large molariform, yellow: caniniform, pink: sharply pointed teeth). The number in brackets indicates the position of each tooth type on the plate.

The upper left plate (ULP), with a toothed surface of 24.4 mm^2^, measures 8 mm (length), 5.6 mm (width) × 5.4 mm (height) and brings 15 teeth (Figure 2b,d,f).

**FIGURE 3:**
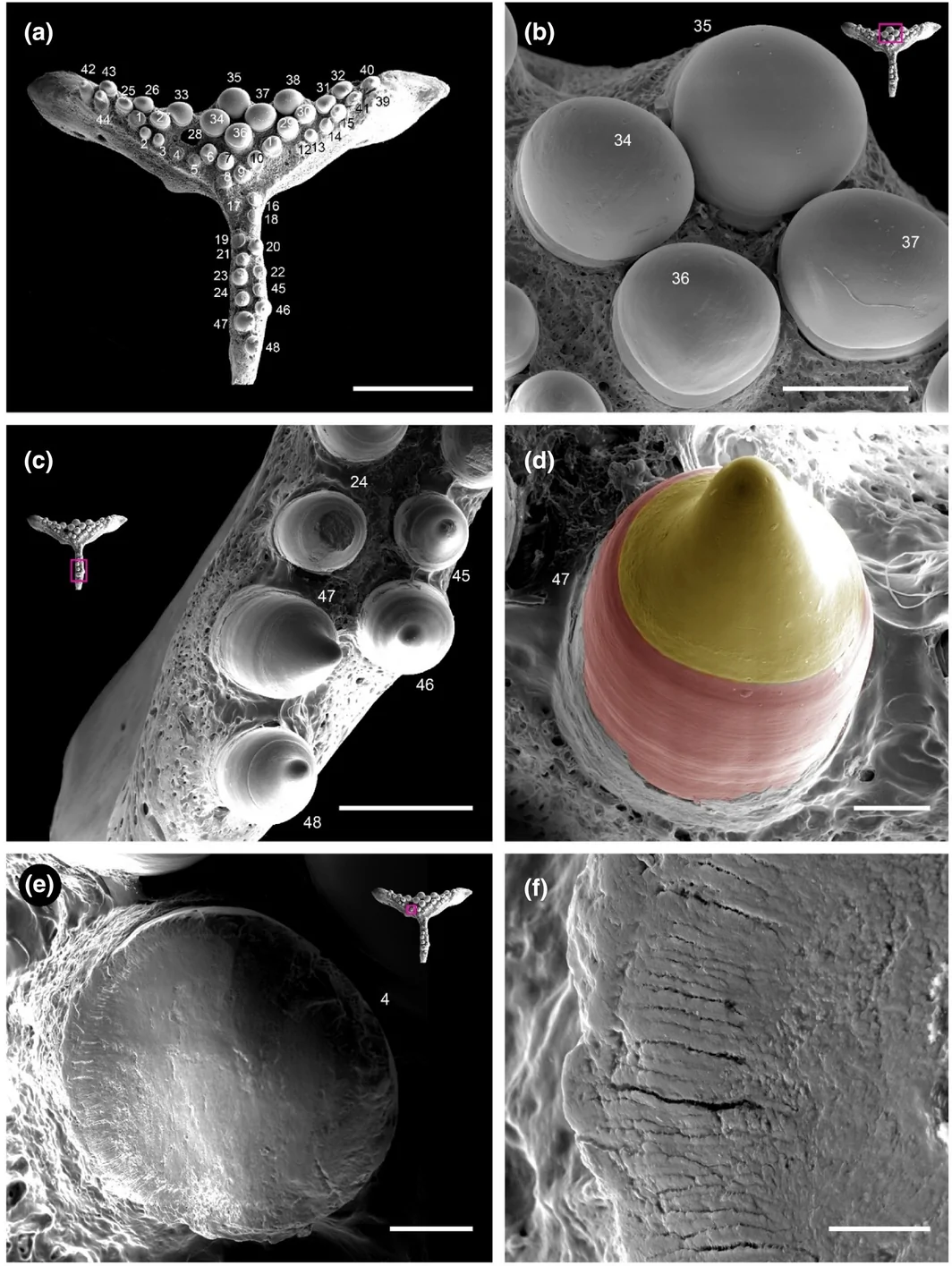
Scanning electron microscope photographs of the trilobate lower plate. (a) Entire plate. (b) Magnification of the central region showing the four largest molariform teeth. (c) Lower region of the plate bearing caniniform teeth. (d) Digitally recoloured micrographs of the caniniform teeth (n. 47) showing the cap acrodin (yellow) and the collar acrodin (red). (e–f) Proximal region of a broken tooth (n. 4) showing short, folded lines resembling weakly developed plicidentine. The pink frame marks the location of the magnified areas on the lower plate. Scale bars: a: 5 mm; b, c: 1 mm; d, e: 200 μm; f: 50 μm.

The LTP is tau‐shaped and characterized by a large and flat, semi‐transparent anterior keel (Figure S1). The plate measures 17.7 mm (distance between horns) × 11.9 mm (axis between keel and tooth 39) × 8.5 mm (height) and shows a toothed area of 40.3 mm^2^ with 48 discernible and 3 worn‐down teeth (Figure 3, Table 1). Some caniniform teeth were broken; in contrast, the molariform teeth were intact.

**FIGURE 4:**
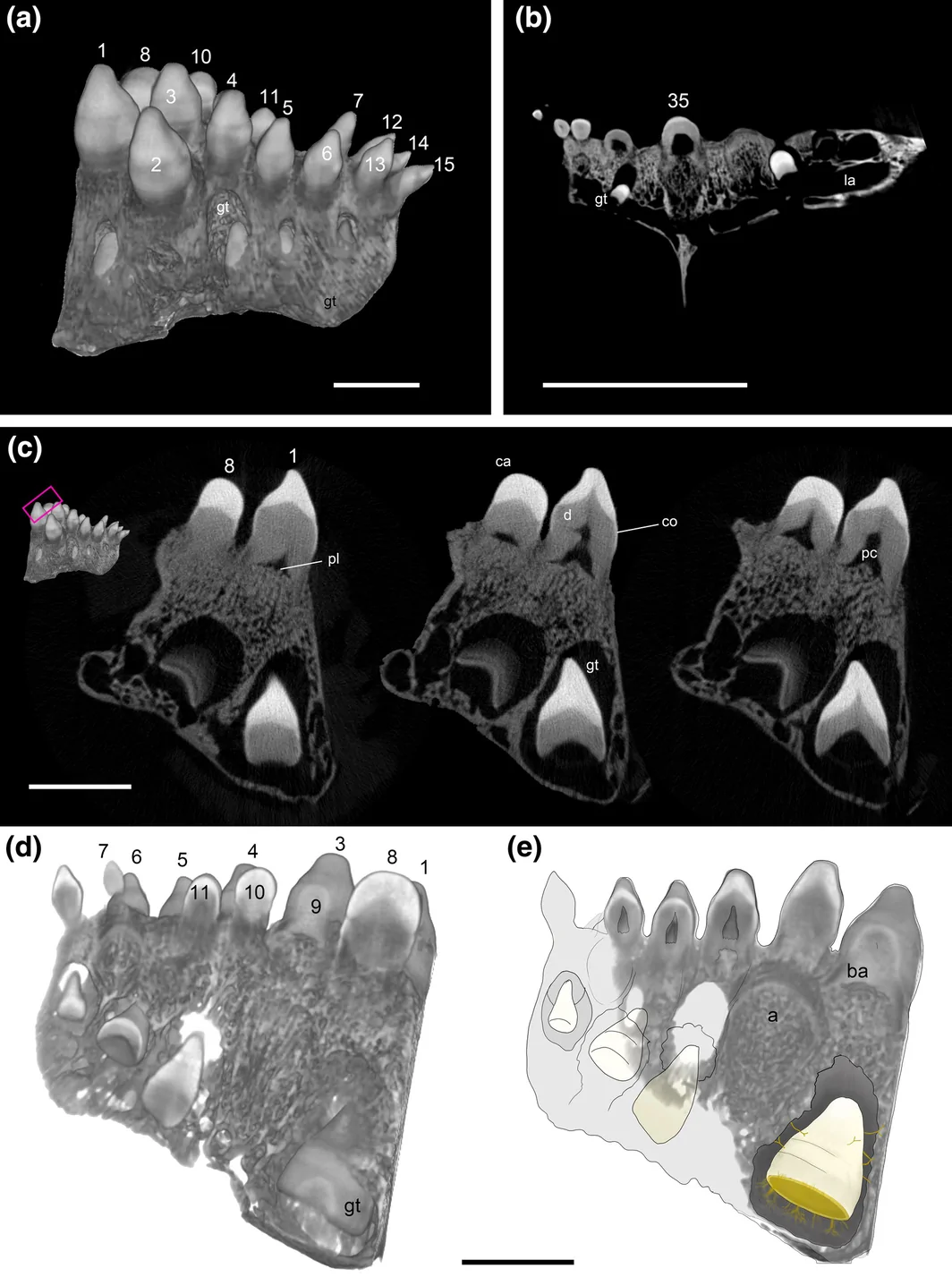
Micro‐computed tomography (micro‐CT) analysis. (a) 3D reconstruction of the upper left plate (ULP), with germ teeth (gt) visible through holes in the dentigerous bone. (b) Virtual sections of the trilobate lower plate revealing the developing germ teeth beneath the plate surface; the smaller germ tooth (gt) is likely emerging from a lacuna (la) running through the basal part of the plate. (c) Sequential slices of the posterior side of the ULP highlighting the largest caniniform and molariform teeth. A presumable presence of plicidentine (pl) is noted on the basal region of the teeth. (d, e) Two sequential micro‐CT virtual slices of the upper left plate. The superimposed interpretative drawing in panel (e) indicates the position of developing caniniform tooth germs within bone crypts. The successional teeth lack attachment and are anchored to the crypt wall by thin collagenous ligaments. a, alveolus; ba, processes likely corresponding to the bone of attachment; ca, cap acrodin; co, collar acrodin; d, dentine; pc, pulp cavity. Scale bars: a: 2 mm; b: 1 cm; c, d, e: 1 mm.

Regardless of the used investigation technique, all teeth exhibited an opaquer basal region and a translucent cap (Figures 2, 3, 4) clearly distinguishable, likely corresponding to the collar acrodin and cap acrodin, respectively (Houée et al., 2025), also called collar enameloid and cap enameloid (Sasagawa et al., 2009). While the cap is smooth, the collar shows parallel striations on its outer surface. The basalmost region of the teeth is covered with spongy bone and soft tissue.

The tips of the caniniform teeth of both the URP and ULP are directed towards the fish's mouth, while the sharpest teeth are positioned at the extremities (Figure 2a–f). One caniniform tooth lay into a depression on the URP (n. 5, Figure 2a) and exhibited slight mobility. It appeared to be loosely anchored to the dentigerous bone through a cylinder of spongy bone permeated with collagenous tissue. Faint vertical parallel incisions were observed on the basal part of this tooth. Upon extraction of the tooth using forceps, examination of its inferior surface revealed the inner pulp cavity and the presence of connective tissue responsible for anchoring the tooth to a cup‐like pit in the alveolar bone (alveolae, Germain & Meunier, 2020). Faint incisions were discernible on the inner walls of the pulp cavity at the base of the tooth.

In the LTP, the molariform teeth are located centrally on the triradiate plate, with the remaining teeth gradually increasing in sharpness towards the vertices (Figure 3a–d). The teeth on the distal extremities of the larger arms of the LTP tend to become elliptical in shape. A broken tooth on the LTP (n. 4, Figure 3e,f) exhibited radially arranged fissures along its inner margin, extending for approximately 50–70 μm.

Virtual sections obtained via micro‐CT revealed the compact structure of the teeth and the porous matrix of the underlying spongy bone. The alveolae, together with the surrounding dentigerous bone, consisted of trabeculate longitudinal canals (Figure 4a–e), resembling the coenosteum (i.e., the calcareous skeleton) of scleractinian corals. The basal region of the functional teeth exhibited two elongated processes, which are presumed to anchor the tooth to the dentigerous bone (Figure 4e).

Micro‐CT of both upper and lower plates unveiled the presence of germ teeth developing vertical into crypts within the dentigerous bone, and corresponding to the locations of the functional teeth, including those firmly attached.

Some of these germ teeth lacked attachment and were also visible through lateral holes present on one side of the ULP (Figures 2f and 4a). One of the hidden caniniform teeth was suspended within the crypts and secured by a web of collagenous bands (Figure 4e). Its basal part was trunked and sealed by a collagenous lid concealing the pulp cavity.

EDS highlighted the average atomic concentration of elements across multiple measurement points on teeth (Supplementary Analysis 1). The principal element identified was Calcium (19.6%), Phosphorous (11.9%), Carbon (28.2%) and Oxygen (38.9%), with a Ca/P ratio of 1.65.

## 4 DISCUSSION

This work first analyses the ultrastructure of the pharyngeal plates of *L. viridis*, thereby contributing to knowledge about a species living in protected habitats (i.e., rocky reefs, Habitat Directive code: 1170, and *Posidonia* meadows, code: 1120) and underscores a significant gap in the trophic ecology of Mediterranean Labridae.

The weak infolding present at the base of the teeth of *L. viridis* and just discernible in the pulp cavity likely is the simplest form of plicidentine (simplexodont plicidentine, Germain et al., 2016). The primary function of plicidentine is to enhance the tooth attachment in predatory fish, although high variability in the development of dentine folds has been reported among vertebrates (Maxwell et al., 2011). Weakly developed plicidentine has been observed in both freshwater and marine fish (Meunier et al., 2015) and was recently reported in gilthead sea breams (Sparidae, Germain & Meunier, 2020) and parrot fish (Labridae, Scarinae, Viviani et al., 2022). Signs of breakage or worn‐down teeth (microspalling, Carr et al., 2006) were observed in our sample, indicating compressive stress, while the parallel layers on the tooth crown may function as shock absorbers.

The elements identified by EDS analysis (Supplementary Analysis 1) likely combine to form Hydroxyapatite (HAp), which exhibits a Ca/P ratio of about 1.67 (Kareem et al., 2024), consistent with that estimated for our samples (1.65). HAp is the principal mineral component of teeth and is responsible for the formation of the hardest mineralized tissues in fish (enameloid, Sasagawa et al., 2009). These findings support the hypothesis that plicidentine may occur in durophagous fish such as *L. viridis* and, together with HAp, likely contributes to reinforcing teeth subjected to considerable mechanical forces (Viviani et al., 2022).

Four main types of tooth attachment have been described for teleost fish (Fink, 1981) and recently revised by Williams et al. (2022). The techniques employed demonstrated both tooth replacement and anchorage to alveolae, a condition termed gomphosis, in contrast to ankylosis, which refers to the permanent attachment of teeth to the bone (Rosa et al., 2021). In our specimen, the two processes visible at the base of functional teeth in virtual sections (Figure 4e) may correspond to the bone of attachment (Rosa et al., 2021), also referred to as pedicels (Leuzinger et al., 2020). These pedicels were not observed in germ teeth, indicating that they form only once the successor tooth reaches its final position.

Due to the limitations of the applied techniques, we cannot provide many details on the attachment modality; nevertheless, we may speculate that the attachment in the studied fish represents Type I (Fink, 1981), given the occurrence of a cylinder of spongy bone surrounding the base of the shedding tooth. High‐resolution Micro‐CT or transmission electron microscopy could confirm the presence of plicidentine, clarify the mode of tooth attachment and shed light on the function of the parallel layers on the collar acrodin.

A plethora of tooth replacement modalities were reported for teleost fishes (Huysseune & Witten, 2024; Leuzinger et al., 2020). Based on our observations, every functional tooth of *L. viridis* is replaced by an underlying tooth germ (one‐for‐one replacement; Figure 4a–e; Tucker & Fraser, 2014). Tooth replacement is intraosseous like in *L. bergylta* (Collins & Underwood, 2021), characterized by germ teeth developing inside crypts of the dentigerous bone (Williams et al., 2022). Our images show teeth at different heights within each crypt, suggesting asynchronous and potentially continuous replacement (polyphyodonty, Leuzinger et al., 2020).

The rate of tooth regeneration in *L. viridis* has not yet been determined; however, it is likely that the replacement cycle is rapid, owing to the durophagous feeding strategy and the need to substitute worn teeth. Tooth turnover may be comparable to that of other teleost fishes, ranging from days to months and varying according to body size and age (Huysseune & Witten, 2024).

Considering that the functional tooth n. 5 was removed with ease from the URP, it is reasonable to hypothesize that both the tooth and the cylinder of spongy bone surrounding the tooth base would have shed within a short time. The ‘floor’ of the alveolus exposed after tooth removal corresponded to the ‘roof’ of the underlying crypt, suggesting that the intraosseous germ tooth development is accompanied by bone remodelling or resorption between the alveolus and the crypt, forming a continuous trench enabling tooth bud migration and eruption (Bemis et al., 2019). In our processed samples, the collagenous web lining the crypts is a remnant of the epithelium surrounding the germ tooth and, considering the discussion in Huysseune and Witten (2024), is probably the successional dental lamina. Although the nature of this epithelium cannot be described with certainty, it likely functions as an elevator, guiding the suspended tooth from the base of the dentigerous plate to the alveolus.

While the germ teeth within the URP and ULP are already vertically oriented, those in the LTP appear to originate within the lacuna, developing horizontally before migrating into the crypts beneath the functional teeth.

In summary, *L. viridis*' pharyngeal jaws exhibit heterodont dentition with unicuspid molariform and caniniform teeth, intraosseous tooth development and a one‐to‐one tooth replacement. The teeth likely have a Type I attachment and show weakly developed plicidentine. No rotation of the germ teeth occurs in the upper plates, whereas a 90° rotation is hypothesized for teeth of the LTP, which are thought to develop horizontally within a lacuna in the lower part of the plate. Vertical ascension of the germ teeth takes place to replace shedding teeth, accompanied by the production of a bone of attachment during the final phases of the replacement process.

The *L. viridis*' PJA differs from that of the congeneric *L. bergylta* which exhibits almost all molariform teeth on both upper and lower plates (Hulsey et al., 2008). This difference may be related to the diets of the two species: *L. bergylta* mainly consumes less mobile, hard‐shelled organisms such as sea urchins, gastropods, decapods (Figueiredo et al., 2005) and bivalves (Deady & Fives, 1995). Consequently, it requires rounded molariform teeth to crush the hard skeletal elements of its prey (Hulsey et al., 2008). Considering the heterodont dentition of *L. viridis*, characterized primarily by caniniform teeth, together with the prey items identified in the gut contents, we can infer that the green wrasse relies on multiple food sources. Gut‐content data from the seven specimens collected in the Gulf of Catania support this interpretation. Among them, four individuals contained identifiable prey items, including crustaceans, amphipods, bivalve fragments and chiton remains, highlighting an opportunistic benthic feeding strategy.

Its molariform, flattened teeth and its caniniform teeth are likely used to crush shelled organisms, whereas the sharper caniniform teeth located at the extremities of the plates may serve to penetrate and retain motile prey. Indeed, considering the shape of the three plates, the URP and ULP only partially overlap with the trilobate plate, and it is likely that the very sharp caniniform teeth do not contact opposing teeth. The occurrence of seagrass fragments in the gut suggests that sharp caniniform teeth may also facilitate a plant‐cutting function (Zeng & Liu, 2011) and rupture of vegetal cells (Burress et al., 2019).

A displacement of *L. viridis* from the core of non‐beaked wrasses cluster was also observed in the dentary phylomorphospace (Evans et al., 2023), highlighting species‐level morphological divergence and functional specialization of lower oral jaws.

Further studies could provide a more complete spectrum of intrageneric variability in *Labrus*, including divergence in oral and pharyngeal jaws as well as intraspecific variations related to sex, size, phenotypic plasticity and adaptability to different environments.

### 4.1 Implications for conservation

The remarkable diversity in the shape and size of pharyngeal jaws in teleost fish, together with variations in tooth morphology, number and arrangement (Burress, 2016; Germain & Meunier, 2020; Raguin et al., 2020; Williams et al., 2022), reflects the finely tuned adaptations of the PJA to a mosaic of trophic niches (Conith & Albertson, 2021). The differences between the PJA of *L. viridis* and *L. bergylta* indicate that high morphofunctional variability may occur within Mediterranean Labridae, in relation to both ecological requirements and prey availability in the fish habitat.

Our results highlight the importance of continuing to compare PJAs and trophic strategies among Mediterranean Labridae. Labridae fish are recognized as indicators of environmental quality (Hinz et al., 2024). When combined with data on other biological traits, patterns of functional morphology become a powerful tool for evaluating links between animals and their habitats, which is crucial in the face of environmental change (De Meyer et al., 2020). Morphofunctional studies help define ecological niche boundaries, assessing phenotypic plasticity and detecting overlaps with potential competitors (Schoenfuss & Blob, 2007), including alien wrasses exploiting similar trophic guilds (Kleitou et al., 2025). This study therefore provides a reference point for linking the morphofunctional traits to the dietary role of *L. viridis*, encouraging new investigations into the morphology of the PJA and trophic dynamics of other Mediterranean wrasses.

## AUTHOR CONTRIBUTIONS

GZ first recorded the pharyngeal jaws. CGDC and GZ conceived the research idea. CGDC wrote the first draft and produced electronic microscope micrographs and tables. GZ collected the samples and created the artworks. FT performed the analyses on the specimens captured in the Gulf of Catania and contributed to the writing and revision of the manuscript. All the authors read and revised the manuscript.

## FUNDING INFORMATION

The current project was funded under the National Recovery and Resilience Plan (NRRP), Mission 4, Component 2, Investment 1.4—Call for tender No. 3138 of 16 December 2021, rectified by Decree n.3175 of 18 December 2021, of the Italian Ministry of University and Research funded by the European Union—NextGenerationEU; Award Number: Project code CN_00000033, Concession Decree No. 1034 of 17 June 2022 adopted by the Italian Ministry of University and Research, CUP D33C22000960007, Project title ‘National Biodiversity Future Center—NBFC’.

## CONFLICT OF INTEREST STATEMENT

The authors declare no conflicts of interest.

## Supporting information


**Data S1.** Supplementary analysis 1: Energy‐dispersive X‐ray spectroscopy (EDS, OXFORD INCAx‐act) showing the elements in the pharyngeal plates.

**Figure S1.** Photos of the dissected fish and details of the pharyngeal jaws.

**Table S1.** Details of the occurrence record and collection of *Labrus viridis*.

## Data Availability

Data are fully available in the main text and Supporting Information.
