# Supplementary material for: The jaws in the jaws: Morphofunctional analysis of the pharyngeal plates of Labrus viridis Linnaeus, 1758 (Teleostei, Labridae)
Source: J Fish Biol. 2026 Jun 14;109(3):1528–39. doi: 10.1111/jfb.70536 (PMC13602090; doi:10.1111/jfb.70536)
Supplement: Supplementary file 1 [file JFB-109-1528-s001.docx]

**Supplementary Figure 1: photos of the dissected fish and details of the pharyngeal jaws**

Dissection of *Labrus viridis* (Linneo, 1758) – Upper and lower plates

Lower trilobate plate
