# Supplementary material for: The jaws in the jaws: Morphofunctional analysis of the pharyngeal plates of Labrus viridis Linnaeus, 1758 (Teleostei, Labridae)
Source: J Fish Biol. 2026 Jun 14;109(3):1528–39. doi: 10.1111/jfb.70536 (PMC13602090; doi:10.1111/jfb.70536)
Supplement: Supplementary file 2 [file JFB-109-1528-s003.docx]

S3 - Energy-dispersive X-ray spectroscopy (EDS)

ULP: Upper Left Plate

URP: Upper Right Plate

#: tooth number

**Supplementary Analysis 1:** Energy-dispersive X-ray spectroscopy (EDS, OXFORD INCAx-act) showing the elements in the pharyngeal plates

**ULP #8**

Page 1 / 13

1. Page

1. Page

1. Page

```
Spectrum: Acquisition

Element     Series  unn. C norm. C Atom. C Error (1 Sigma)
                    [wt.%]  [wt.%]  [at.%]          [wt.%]
----------------------------------------------------------
```

Calcium	K-series 19.37	28.39	13.46	0.60

```
Phosphorus K-series  10.14   14.87    9.12            0.41
Oxygen     K-series  18.68   27.38   32.51            2.42
Iron       K-series   0.31    0.45    0.15            0.04
Carbon     K-series  18.86   27.65   43.73            2.45
Sodium     K-series   0.67    0.97    0.81            0.07
Magnesium  K-series   0.19    0.28    0.22            0.04
----------------------------------------------------------
             Total:  68.21  100.00  100.00
```

Page 3 / 4

3. Page

3. Page

3. Page

```
Spectrum: Acquisition

Element     Series  unn. C norm. C Atom. C Error (1 Sigma)
                    [wt.%]  [wt.%]  [at.%]          [wt.%]
----------------------------------------------------------
Calcium    K-series  16.96   16.53    6.79            0.53
Phosphorus K-series   8.68    8.47    4.50            0.36
Oxygen     K-series  38.26   37.30   38.38            4.50
Iron       K-series   0.33    0.32    0.09            0.04
Carbon     K-series  36.81   35.89   49.19            4.34
Sodium     K-series   1.24    1.21    0.86            0.11
Magnesium  K-series   0.29    0.28    0.19            0.04
----------------------------------------------------------
             Total: 102.57  100.00  100.00
```

Page 4 / 4

4. Page

4. Page

4. Page

```
Spectrum: Acquisition

Element     Series  unn. C norm. C Atom. C Error (1 Sigma)
                    [wt.%]  [wt.%]  [at.%]          [wt.%]
----------------------------------------------------------
Calcium    K-series  39.26   66.69   55.54            1.22
Phosphorus K-series  15.14   25.73   27.72            0.62
Oxygen     K-series   1.04    1.77    3.69            0.48
Iron       K-series   0.84    1.43    0.85            0.07
Carbon     K-series   2.58    4.39   12.19            0.80
Sodium     K-series   0.00    0.00    0.00            0.00
Magnesium  K-series   0.00    0.00    0.00            0.00
----------------------------------------------------------
             Total:  58.87  100.00  100.00
```

Page 5 / 5

5. Page

5. Page

5. Page

```
Spectrum: Acquisition

Element     Series  unn. C norm. C Atom. C Error (1 Sigma)
                    [wt.%]  [wt.%]  [at.%]          [wt.%]
----------------------------------------------------------
Calcium    K-series  19.87   22.27   10.23            0.62
Phosphorus K-series  10.06   11.27    6.70            0.41
Oxygen     K-series  38.88   43.58   50.16            4.53
Iron       K-series   0.58    0.65    0.21            0.05
Carbon     K-series  18.15   20.34   31.19            2.31
Sodium     K-series   1.40    1.56    1.25            0.12
Magnesium  K-series   0.29    0.33    0.25            0.04
----------------------------------------------------------
             Total:  89.22  100.00  100.00
```

Page 6 / 6

6. Page

6. Page

6. Page

```
Spectrum: Acquisition

Element     Series  unn. C norm. C Atom. C Error (1 Sigma)
                    [wt.%]  [wt.%]  [at.%]          [wt.%]
----------------------------------------------------------
Calcium    K-series  27.62   47.02   30.27            0.85
Phosphorus K-series  14.64   24.91   20.75            0.58
Oxygen     K-series   9.34   15.89   25.63            1.47
Iron       K-series   0.81    1.38    0.64            0.06
Carbon     K-series   6.07   10.33   22.19            1.07
Sodium     K-series   0.23    0.39    0.44            0.04
Magnesium  K-series   0.04    0.07    0.07            0.00
----------------------------------------------------------
             Total:  58.75  100.00  100.00
```

Page 7 / 7

7. Page

7. Page

7. Page

```
Spectrum: Acquisition

Element     Series  unn. C norm. C Atom. C Error (1 Sigma)
                    [wt.%]  [wt.%]  [at.%]          [wt.%]
----------------------------------------------------------
Calcium    K-series  25.17   32.29   17.02            0.78
Phosphorus K-series  12.68   16.27   11.10            0.51
Oxygen     K-series  28.86   37.02   48.88            3.55
Iron       K-series   0.49    0.63    0.24            0.05
Carbon     K-series   9.38   12.04   21.17            1.38
Sodium     K-series   1.07    1.38    1.26            0.10
Magnesium  K-series   0.29    0.37    0.32            0.04
----------------------------------------------------------
             Total:  77.95  100.00  100.00
```

Page 8 / 8

8. Page

8. Page

8. Page

```
Spectrum: Acquisition

Element     Series  unn. C norm. C Atom. C Error (1 Sigma)
                    [wt.%]  [wt.%]  [at.%]          [wt.%]
----------------------------------------------------------
Calcium    K-series  31.89   55.54   39.28            0.98
Phosphorus K-series  14.48   25.23   23.09            0.58
Oxygen     K-series   6.60   11.50   20.38            1.19
Iron       K-series   0.30    0.53    0.27            0.05
Carbon     K-series   4.12    7.18   16.95            0.84
Sodium     K-series   0.01    0.03    0.03            0.00
Magnesium  K-series   0.00    0.00    0.00            0.00
----------------------------------------------------------
             Total:  57.42  100.00  100.00
```

Page 8 / 8

8. Page

8. Page

8. Page

```
Spectrum: Acquisition

Element     Series  unn. C norm. C Atom. C Error (1 Sigma)
                    [wt.%]  [wt.%]  [at.%]          [wt.%]
----------------------------------------------------------
Calcium    K-series  26.14   34.04   18.20            0.81
Phosphorus K-series  13.20   17.19   11.89            0.53
Oxygen     K-series  26.53   34.55   46.26            3.33
Iron       K-series   0.12    0.15    0.06            0.03
Carbon     K-series   9.47   12.33   21.99            1.40
Sodium     K-series   1.06    1.38    1.29            0.10
Magnesium  K-series   0.28    0.36    0.32            0.04
----------------------------------------------------------
             Total:  76.80  100.00  100.00
```

Page 9 / 9

9. Page

9. Page

9. Page

```
Spectrum: Acquisition

Element     Series  unn. C norm. C Atom. C Error (1 Sigma)
                    [wt.%]  [wt.%]  [at.%]          [wt.%]
----------------------------------------------------------
Calcium    K-series  22.76   29.84   14.55            0.71
Phosphorus K-series  11.65   15.27    9.64            0.47
Oxygen     K-series  21.60   28.32   34.59            2.91
Iron       K-series   0.48    0.63    0.22            0.05
Carbon     K-series  18.64   24.43   39.75            2.60
Sodium     K-series   0.91    1.19    1.01            0.09
Magnesium  K-series   0.23    0.31    0.25            0.04
----------------------------------------------------------
             Total:  76.27  100.00  100.00
```

**ULP #6**

Page 10 / 13

10. Page

10. Page

10. Page

```
Spectrum: Acquisition

Element     Series  unn. C norm. C Atom. C Error (1 Sigma)
                    [wt.%]  [wt.%]  [at.%]          [wt.%]
----------------------------------------------------------
Calcium    K-series  32.14   32.79   17.27            0.99
Phosphorus K-series  14.37   14.66    9.99            0.57
Oxygen     K-series  39.20   39.98   52.75            4.93
Iron       K-series   0.58    0.60    0.23            0.06
Carbon     K-series  10.25   10.46   18.38            1.62
Sodium     K-series   1.19    1.21    1.11            0.11
Magnesium  K-series   0.31    0.32    0.27            0.05
----------------------------------------------------------
             Total:  98.04  100.00  100.00
```

Page 11 / 12

11. Page

11. Page

11. Page

```
Spectrum: Acquisition

Element     Series  unn. C norm. C Atom. C Error (1 Sigma)
                    [wt.%]  [wt.%]  [at.%]          [wt.%]
----------------------------------------------------------
Calcium    K-series  33.10   33.03   17.56            1.02
Phosphorus K-series  13.83   13.80    9.49            0.55
Oxygen     K-series  45.31   45.21   60.20            5.54
Iron       K-series   0.00    0.00    0.00            0.00
Carbon     K-series   6.39    6.38   11.31            1.10
Sodium     K-series   1.01    1.00    0.93            0.09
Magnesium  K-series   0.59    0.59    0.52            0.06
----------------------------------------------------------
             Total: 100.22  100.00  100.00
```

Page 12 / 12

12. Page

12. Page

12. Page

```
Spectrum: Acquisition

Element     Series  unn. C norm. C Atom. C Error (1 Sigma)
                    [wt.%]  [wt.%]  [at.%]          [wt.%]
----------------------------------------------------------
Calcium    K-series   4.39    5.20    1.84            0.16
Phosphorus K-series   3.14    3.72    1.71            0.15
Oxygen     K-series  29.10   34.50   30.61            3.59
Iron       K-series   0.12    0.14    0.04            0.04
Carbon     K-series  46.32   54.91   64.89            5.36
Sodium     K-series   0.89    1.05    0.65            0.09
Magnesium  K-series   0.39    0.46    0.27            0.05
----------------------------------------------------------
             Total:  84.35  100.00  100.00
```

Page 13 / 13

13. Page

13. Page

13. Page

```
Spectrum: Acquisition

Element     Series  unn. C norm. C Atom. C Error (1 Sigma)
                    [wt.%]  [wt.%]  [at.%]          [wt.%]
----------------------------------------------------------
Calcium    K-series  25.62   24.98   12.41            0.79
Phosphorus K-series  13.73   13.39    8.60            0.55
Oxygen     K-series  51.37   50.09   62.31            6.03
Iron       K-series   0.23    0.22    0.08            0.04
Carbon     K-series   8.85    8.63   14.30            1.35
Sodium     K-series   1.86    1.81    1.57            0.15
Magnesium  K-series   0.90    0.88    0.72            0.08
----------------------------------------------------------
             Total: 102.57  100.00  100.00
```
